# Tuberculosis-Related Hospitalization According to Comorbidity Burden: A Retrospective Single-Center Cohort Study

**DOI:** 10.3390/tropicalmed11070193

**Published:** 2026-07-10

**Authors:** Oh Beom Kwon, Yeonjeong Heo, Da Hye Moon, Woo Jin Kim, Seung-Joon Lee, Seon-Sook Han

**Affiliations:** Division of Pulmonary and Critical Care Medicine, Department of Internal Medicine, Kangwon National University Hospital, Chuncheon 24289, Republic of Korea; obkwon@kangwon.ac.kr (O.B.K.); yonjong1954@naver.com (Y.H.); ansekgo@naver.com (D.H.M.); pulmo2@kangwon.ac.kr (W.J.K.); medfman@kangwon.ac.kr (S.-J.L.)

**Keywords:** tuberculosis, hospitalization, Charlson comorbidity index

## Abstract

Tuberculosis (TB) remains a major infectious disease associated with substantial healthcare burdens. Although previous studies mainly focused on mortality and treatment outcomes, factors associated with TB-related hospitalization have not been sufficiently investigated. The aim of our study was to evaluate clinical factors associated with TB-related hospitalization in patients with TB. Patients diagnosed with TB at Kangwon National University Hospital between January 2024 and December 2025 were included in our study. The primary outcome was TB-related hospitalization during follow-up after TB diagnosis. Hospitalization-free probability was analyzed using Kaplan–Meier analysis, and Cox proportional hazards regression analysis was performed to identify factors associated with hospitalization risk. A total of 81 patients were included; 63 patients were in the outpatient group and 18 patients were in the hospitalization group. The median time to hospitalization was 36 days. The Charlson comorbidity index (CCI) was significantly higher in the hospitalization group (5.78 ± 2.88 vs. 4.16 ± 2.22, *p* = 0.038). Patients with CCI values ≥ 5 showed significantly lower hospitalization-free probability during the 180-day follow-up period (log-rank *p* = 0.013). In multivariable Cox hazards regression analysis, both serum creatinine (hazard ratio (HR), 1.599; 95% confidence interval (CI), 1.057–2.419; *p* = 0.026) and CCI (HR, 1.221; 95% CI, 1.018–1.466; *p* = 0.032) were significantly associated with TB-related hospitalization. A higher comorbidity burden may help identify patients at risk for TB-related hospitalization.

## 1. Introduction

Tuberculosis (TB) is an infectious disease and remains a major global public health issue with a high disease burden [1]. In 2024, the number of TB cases in the Republic of Korea (ROK) was 17,944 (35.2 cases per 100,000 population), continuing a decreasing trend over the past decade [2]. However, the proportion of medical aid recipients among patients with TB increased from 2.9% in 2023 to 11.3% in 2024, suggesting an increasing socioeconomic burden associated with the disease [2]. Most patients with TB are managed in the outpatient setting. However, hospitalization is often required in patients with severe disease, treatment-related complications, or significant comorbid conditions. A previous study has shown that hospitalization is associated with poor clinical outcomes, and the proportion of patients admitted through the emergency room or intensive care unit was higher among non-survivors than among survivors [3]. Because hospitalization is associated with poor clinical outcomes and increased healthcare utilization, early identification of patients at increased risk for hospitalization may facilitate closer monitoring during TB treatment and help reduce the socioeconomic burden of TB.

Nutritional status and comorbidities were identified as risk factors associated with mortality in patients with pulmonary TB [4]. Nutrition status was assessed by nutrition risk score which consists of body mass index, serum albumin, serum cholesterol, and lymphocyte count [5]. Kim et al. have shown that mortality was associated with decreased hemoglobin, lymphocyte, albumin and cholesterol. Independent predictors associated with mortality were blood urea nitrogen and admission during treatment of TB [3]. A retrospective cohort study from Australia suggested that all-cause mortality was associated with renal impairment in patients with TB in a low TB prevalence [6]. Anti-TB drug-induced liver injury contributes to longer hospital stays and increased economic burden [7]. An inflammatory biomarker, C-reactive protein (CRP) was associated with mortality in critically ill patients with TB [8]. High white blood cell (WBC) count and low lymphocyte proportions were associated with the risk of treatment failure [9]. The neutrophil-to-lymphocyte ratio (NLR) represents the severity of inflammation and is known to predict outcomes in infectious diseases. Pulmonary TB patients with a higher NLR had a worse short-term survival rate compared to those with a lower NLR [10]. Moreover, a positive acid-fast bacilli (AFB) smear result was associated with TB-related death [11].

Previous studies mainly focused on mortality and treatment outcomes. However, relatively limited attention has been directed toward TB-related hospitalization. A nationwide study from Spain reported 29,942 TB-related hospitalizations between 2012 and 2020, corresponding to an annual hospitalization rate of 7.1 admissions per 100,000 population [12]. In addition, TB-related hospitalization is associated with substantial inpatient healthcare utilization and medical costs [13]. Therefore, early identification of patients at increased risk for hospitalization may help clinicians optimize monitoring strategies and potentially reduce the healthcare burden during TB treatment. The demographic characteristics of patients with TB are changing. In ROK, the proportion of patients aged 65 years or older has steadily increased over the past decade [14]. Older patients with TB frequently have multiple chronic medical conditions, and multimorbidity has become increasingly common in this population. Because comorbid diseases may influence treatment tolerance, clinical course, and healthcare utilization, assessment of comorbidity burden has become an important component of TB management. Furthermore, the growing burden of chronic diseases in aging populations highlights the need to better understand the impact of comorbidities on clinical outcomes in patients with TB.

The Charlson comorbidity index (CCI) is a widely used measure of comorbidity burden and has been associated with adverse clinical outcomes, such as mortality [4], in patients with TB. The CCI incorporates both age and underlying chronic diseases and provides a simple method for evaluating overall comorbidity burden. Although previous studies have demonstrated an association between higher CCI scores and mortality [4], its relationship with TB-related hospitalization has not been well characterized. We hypothesized that a higher comorbidity burden, as assessed by the CCI, would be associated with an increased risk of TB-related hospitalization. Accordingly, this study evaluated the association between comorbidity burden and TB-related hospitalization in patients with TB.

## 2. Materials and Methods

### 2.1. Patients

**Figure 1:**
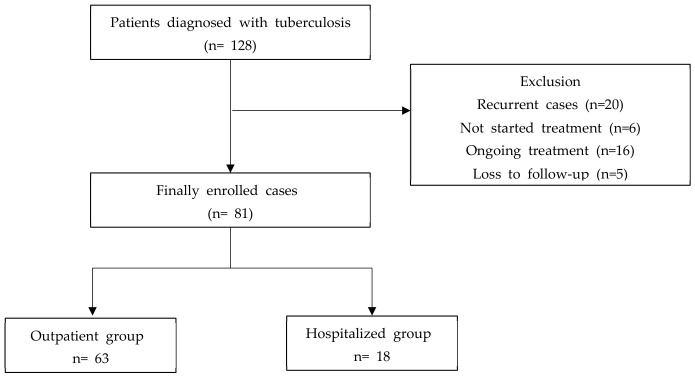
Flow chart of patient selection and study population.

Patients who were diagnosed with TB at Kangwon National University Hospital between January 2024 and December 2025 were screened for inclusion in this study. Kangwon National University Hospital is a regional referral hospital in Gangwon Province and provides care to patients throughout the region. Patients with TB are frequently referred from local hospitals and clinics, while others are initially diagnosed and treated at Kangwon National University Hospital. The diagnosis of TB was established according to the previous study [15]. Microbiological confirmation was defined as positive microbiological tests, including polymerase chain reaction, Xpert MTB/RIF Ultra (Cepheid, Sunnyvale, CA, USA), or mycobacterial culture. Histopathological confirmation was based on pathological findings consistent with TB. In the absence of microbiological or histopathological confirmation, TB was diagnosed based on clinical and radiological findings. TB pleurisy was diagnosed based on lymphocyte-predominant pleural effusion with adenosine deaminase levels ≥ 40 U/L [16]. The inclusion criteria were patients over 18 years of age and diagnosed with TB. A total of 128 patients were extracted from the electrical medical records (EMR). As shown in Figure 1, the exclusion criteria were as follows: (1) recurrent TB, (2) ongoing anti-TB treatment, (3) no anti-TB treatment initiation and (4) loss to follow-up. After applying the exclusion criteria, a total of 81 patients were enrolled in this study.

### 2.2. Variables and Outcome Definition

Demographic, laboratory, and microbiological data were collected from EMR at the time of TB diagnosis. Demographic variables included age and sex. Laboratory variables included WBC count, neutrophil count, lymphocyte count, NLR, hemoglobin, creatinine, albumin, aspartate-aminotransferase (AST), alanin-aminotransferase (ALT), total bilirubin, CRP, and uric acid levels. Creatinine was used to represent kidney functions and ALT was used to represent liver function. Lymphocyte and albumin were used to represent nutrition status. Total WBC, neutrophil, NLR and CRP were used to represent the severity of inflammation. CCI was used to represent comorbidity. AFB smear results, location of TB and all-cause mortality were also collected. Analyses were performed using available data, and observations with missing values were excluded from the relevant analyses.

The primary outcome of this study was hospitalization due to TB-related symptoms within the follow-up period after TB diagnosis. TB-related symptoms included respiratory symptoms (e.g., fever, dyspnea, hemoptysis [15]), treatment-related complications and general weakness. The cause of hospitalization was determined through review of the EMR. Patients who were already hospitalized at the time of TB diagnosis were not considered as having a hospitalization event, because the aim of this study was to evaluate subsequent TB-related admissions following diagnosis.

### 2.3. Statistical Analysis

Continuous variables are presented as mean ± standard deviation and categorical variables are presented as numbers with percentages. Patients were grouped into two groups: hospitalized group and outpatient group. The normality of continuous variables was assessed using the Shapiro–Wilk test. Student’s *t*-test was used for the comparison of continuous variables between the two groups and a Chi-squared test was performed for the comparison of categorical variables. Fisher’s exact test was applied when the expected frequency in any cell was less than 5.

Hospitalization-free probability was evaluated using Kaplan–Meier survival analysis and compared with log-rank test. The study population was divided into two groups according to the median CCI value. Since the median CCI value was 4 points, patients with 5 points or higher were assigned to the high CCI group, while those with scores below 5 points were assigned to the low CCI group. Patients who were treated only at the outpatient department during the follow-up were censored at 180 days after TB diagnosis.

Factors associated with TB-related hospitalization were analyzed using Cox proportional hazards regression models. Hazard ratio (HR) and 95% confidence interval (CI) were analyzed. Variables with *p* < 0.05 in the univariable analysis were included in the multivariable analysis. To prevent model overfitting and adhere to an acceptable event-per-variable ratio, we limited the Cox regression analysis to three variables, reflecting the hospitalization events (*n* = 18). NLR, creatinine and CCI were selected based on their clinical relevance and previously reported associations with adverse outcomes in patients with TB [10,17]. The proportional hazards assumption for the Cox regression model was assessed using Schoenfeld residuals. All analyses were conducted using R version 4.4.2 (R foundation for Statistical Computing, Vienna, Austria).

## 3. Results

### 3.1. Overall Patient Characteristics and Comparison Between Hospitalization and Outpatient Groups

A total of 81 patients were diagnosed with TB during the study period. Among 81 patients, 20 patients were diagnosed with TB during an ongoing hospitalization and were classified into the outpatient group because hospitalization had occurred before TB diagnosis. The final cohort consisted of 63 patients in the outpatient group, and 18 patients were in the hospitalization group. The median time from TB diagnosis to hospitalization was 36 days (interquartile range, 14–116 days). Characteristics of the patients and comparison between the outpatient group and the hospitalization group are presented in Table 1. The mean age was similar between the outpatient and hospitalization group. Although the proportion of males was higher in the hospitalization group, the difference was not statistically significant (*p* = 0.173). Hematologic parameters, including WBC count, neutrophil count, lymphocyte count, hemoglobin level, and platelet count were not significantly different between two groups. Similarly, biochemical parameters, including albumin, AST, ALT, total bilirubin, CRP, and uric acid levels, were not significantly different between the two groups.

CCI was significantly higher in the hospitalization group compared with the outpatient group. Additionally, the proportion of patients with CCI ≥ 5 was higher in the hospitalization group. Although NLR and creatinine levels tended to be higher in the hospitalization group, the difference did not reach statistical significance.

The distribution of individual comorbidities included in the CCI is presented in Supplementary Table S1. Diabetes mellitus (DM) was the most prevalent comorbidity (33.34%), followed by cerebrovascular disease (CVD) (16.05%), and moderate-to-severe chronic kidney disease (9.88%). The methods of TB diagnosis are summarized in Supplementary Table S2. Among the 81 patients, 61 (75.31%) had microbiologically confirmed TB, 6 (7.41%) had histopathological confirmation, and 14 (17.28%) were diagnosed based on clinical and radiological findings. Patients with TB pleurisy were included in the clinical and radiological confirmation group.

### 3.2. Kaplan–Meier Analysis According to Charlson Comorbidity Index

**Figure 2:**
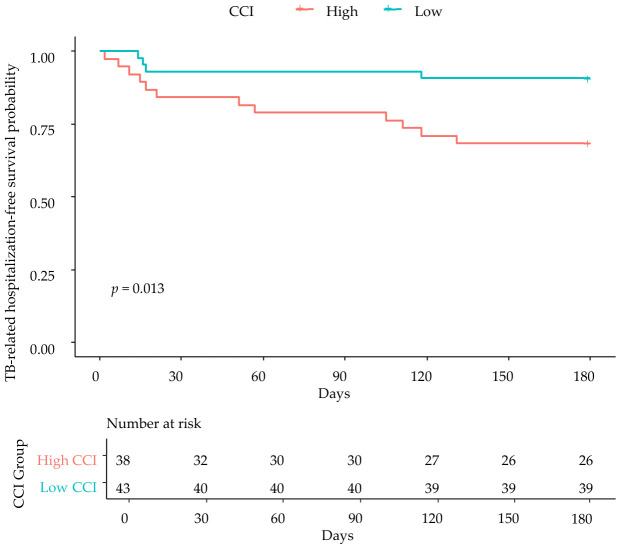
Kaplan–Meier curves of TB-related hospitalization-free probability according to CCI category (CCI ≥ 5 vs. CCI < 5) (log-rank *p* = 0.013). TB, tuberculosis; CCI, Charlson comorbidity index.

Kaplan–Meier analysis revealed a distinct separation in hospitalization-free probabilities when stratified by the median CCI score (4 points). As illustrated in Figure 2, the high CCI group (CCI ≥ 5) showed significantly lower TB-related hospitalization-free probability compared to the low CCI group (CCI < 5). Over the course of follow-up, the high CCI group exhibited an accumulation of admission events, whereas the low CCI group maintained a stable trajectory over time.

### 3.3. Risk Factors for Hospitalization in Tuberculosis Patients

The results of the univariable and multivariable Cox proportional hazards regression analyses are presented in Table 2. The proportional hazards assumption was satisfied based on Schoenfeld residual testing (global test *p* = 0.30). In univariable analysis, higher serum creatinine levels and higher CCI values were associated with an increased risk of TB-related hospitalization. In multivariable analysis, both serum creatinine (HR 1.599, 95% CI 1.057–2.419, *p* = 0.026) and CCI (HR 1.221, 95% CI 1.018–1.466, *p* = 0.032) remained independently associated with TB-related hospitalization. Because the Kaplan–Meier analysis used a CCI cutoff value of 5, we additionally evaluated CCI as a continuous variable in the Cox regression model. The association between CCI and hospitalization remained significant, supporting the robustness of the findings.

## 4. Discussion

In our study, a higher CCI was associated with an increased risk of TB-related hospitalization during follow-up in patients with TB. Patients with CCI ≥ 5 demonstrated significantly lower hospitalization-free probability in Kaplan–Meier analysis. These findings suggest that comorbidity burden may substantially influence the clinical course of TB and the likelihood of subsequent hospitalization. In the Cox proportional hazards regression analysis, each one-point increase in CCI was associated with an approximately 22% increase in the risk of TB-related hospitalization. This finding suggests a possible association between comorbidity burden and hospitalization risk in patients with TB. DM was the most common comorbid condition. DM is a well-established risk factor for TB and has been associated with poor outcomes. Furthermore, poor glycemic control may impair host immune responses, whereas adequate glycemic control has been associated with improved treatment outcomes [18]. CVD was the second most common comorbidity in our cohort. Previous studies have suggested that chronic inflammation associated with TB may promote atherosclerosis and contribute to an increased risk of CVD. Furthermore, patients with TB had a higher incidence of subsequent CVD compared with the matched controls [19]. These findings suggest that management of comorbid conditions is an important component of TB care. Patients with multiple chronic diseases may be more vulnerable to the development of TB and adverse clinical outcomes because of impaired immune function. Therefore, careful monitoring and appropriate management of comorbid conditions may be crucial throughout the course of TB treatment.

Previous studies on TB have mainly focused on mortality and treatment responses [20,21]. In contrast, fewer studies have specifically evaluated hospitalization after TB diagnosis, despite its clinical and socioeconomic importance. Hospital admission in patients with TB often leads to increased medical costs [22], prolonged isolation, and caregiver burden. Identifying patients who are more likely to require hospitalization may help clinicians to organize outpatient monitoring strategies and reduce healthcare burden.

The proportion of patients with TB aged ≥65 years increased from 2011 to 2020 in ROK [14]. The burden of chronic diseases and multimorbidity continues to increase in aging populations [23]. Because CCI incorporates both age and underlying chronic diseases, the overall CCI of patients with TB may also increase over time. Previous study has shown that systematic approach, including nutritional support, patient- and family-centered care and symptom control, is essential when managing chronic critical illness patients [24]. However, data regarding the relationship between comorbidity burden and TB-related hospitalization remain limited. Our findings suggest that comorbidity burden may also play an important role in hospitalization risk during anti-TB treatment. These findings may help identify patients who require closer monitoring during anti-TB treatment.

Hematologic and biochemical markers such as anemia, leukocytosis, lymphopenia, and hypoalbuminemia have been observed in patients with severe TB [15,25]. In our study, hemoglobin and albumin levels were similar between groups. Although statistical significance was not reached, patients in the hospitalized group tended to have higher leukocyte counts and lower lymphocyte counts, resulting in higher NLR. In Cox proportional hazards regression analysis, NLR was not associated with TB-related hospitalization. However, the relatively small number of hospitalization events may have limited the statistical stability of the multivariable model and reduced the ability to fully evaluate the impact of baseline inflammatory markers such as NLR. Previous studies have reported that NLR was associated with an increased risk of mortality in patients with TB [26,27]. Therefore, the potential clinical relevance of NLR should not be underestimated and warrants further investigation in larger cohorts.

In our study, elevated serum creatinine was associated with increased risk of TB-related hospitalization. Previous studies have reported that reduced kidney function was associated with worse TB treatment outcomes [6,17]. Chronic kidney disease is associated with immune dysfunction and often requires multiple medications, increasing the risk of drug–drug interactions during anti-TB treatment. In addition, reduced renal clearance may alter anti-TB drug concentrations. These factors may contribute to unfavorable clinical outcomes [17] and an increased risk of hospitalization. Serum albumin represents the nutrition status of the patient and is known to be an important risk factor for mortality in patients with TB [4]. However, albumin levels were within normal range in both groups, which may explain why albumin was not associated with hospitalization risk.

However, there are several limitations in our study. First, this was a retrospective single-center study with a relatively small sample size and a limited number of hospitalization events. Second, recurrent TB cases were excluded to minimize clinical heterogeneity because previous treatment exposure and residual pulmonary function may affect hospitalization risk. However, this might lead to selection bias. Third, several potentially important confounders, including smoking status, socioeconomic factors, medication adherence, and nutritional status, were not available in our data. Finally, external validation was not performed, and additional multi-center studies are required.

## 5. Conclusions

Higher CCI values were significantly associated with increased TB-related hospitalization during follow-up. Patients with higher comorbidity burden had a substantially lower hospitalization-free probability than those with lower CCI values. These findings suggest that baseline comorbidity status may help identify patients at increased risk for hospitalization and may contribute to reducing healthcare burden through intense monitoring and earlier intervention during TB treatment. Further large-scale, multi-center studies are warranted to validate these relationships and optimize risk-stratified monitoring strategies.

## Figures and Tables

**Table 1:** Characteristics of the patients and comparison between hospitalization group and outpatient group.

	Outpatient (*n* = 63)	Hospitalization (*n* = 18)	*p*-Value
Age (years)	71.65 ± 15.44	72.78 ± 13.10	0.760
Male (%)	38 (60.32)	14 (77.78)	0.173
Total WBC (/μL)	7141.27 ± 2542.52	8088.89 ± 3076.84	0.244
Neutrophil (/μL)	4397.55 ± 2463.22	5399.34 ± 3162.34	0.227
Lymphocyte(/μL)	1520.56 ± 817.16	1264.09 ± 661.51	0.179
NLR	4.16 ± 4.38	5.68 ± 5.32	0.293
Hemoglobin (g/dL)	11.89 ± 1.81	11.94 ± 2.24	0.919
Platelet (×10^3^/μL)	256.40 ± 83.00	291.61 ± 120.00	0.256
Creatinine (mg/dL)	1.02 ± 0.65	1.34 ± 1.26	0.312
Albumin (g/dL)	3.52 ± 0.62	3.51 ± 0.52	0.910
AST (U/L)	44.81 ± 81.18	34.72 ± 23.45	0.388
ALT (U/L)	24.68 ± 22.34	23.67 ± 21.58	0.863
Total bilirubin (mg/dL)	0.65 ± 0.49	0.70 ± 0.30	0.397
CRP (mg/dL)	3.98 ± 4.18	4.89 ± 3.72	0.498
Uric acid (mg/dL)	5.57 ± 3.13	6.54 ± 2.78	0.238
CCI	4.16 ± 2.22	5.78 ± 2.88	0.038
CCI ≥ 5	24 (38.10)	14 (77.78)	0.008
AFB stain	11 (17.46)	2 (11.11)	0.604
Location of TB			0.439
Pulmonary TB (%)	47 (74.60)	11 (61.11)	
Extrapulmonary TB (%)	12 (19.05)	6 (33.33)	
Combined (%)	4 (6.35)	1 (5.56)	
All-cause mortality (%)	13 (20.63)	7 (38.89)	0.113

WBC, white blood cell; NLR, neutrophil-to-lymphocyte ratio; AST, aspartate-aminotransferase; ALT, alanin-aminotransferase; CRP, C-reactive protein; CCI, Charlson comorbidity index; AFB, acid-fast bacilli; TB, tuberculosis.

**Table 2:** Cox proportional hazards regression analysis for factors associated with TB-related hospi-talization.

	Univariable		Multivariable	
	Hazard ratio (95% CI)	*p*-value	Hazard ratio (95% CI)	*p*-value
NLR	0.944 (0.988–1.135)	0.108		
Creatinine (mg/dL)	1.698 (1.123–2.567)	0.012	1.599 (1.057–2.419)	0.026
CCI	1.225 (1.033–1.452)	0.019	1.221 (1.018–1.466)	0.032

TB, tuberculosis; CI, confidence interval; NLR, neutrophil-to-lymphocyte ratio; CCI, Charlson comorbidity index.

## Data Availability

The data presented in this study are available on request from the corresponding author.

## Supplementary Materials

The following supporting information can be downloaded at: https://www.mdpi.com/article/10.3390/tropicalmed11070193/s1. Table S1. Distribution of individual Charlson Comorbidity Index comorbidities among patients with tuberculosis; Table S2. Methods of tuberculosis diagnosis.

## Abbreviations

The following abbreviations are used in this manuscript:

TB	Tuberculosis
ROK	Republic of Korea
CRP	C –reactive protein
WBC	White blood cell
NLR	Neutropihl-to-lymphocyte ratio
AFB	Acid-fast bacilli
CCI	Charlson comorbidity index
EMR	Electrical medical records
AST	Aspartate-aminotransferase
ALT	Alanine-aminotransferase
HR	Hazard ratio
CI	Confidence interval
DM	Diabetes mellitus
CVD	Cerebrovascular disease
