# Supplementary material for: Tuberculosis-Related Hospitalization According to Comorbidity Burden: A Retrospective Single-Center Cohort Study
Source: Trop Med Infect Dis. 2026 Jul 10;11(7):193. doi: 10.3390/tropicalmed11070193 (PMC13417246; doi:10.3390/tropicalmed11070193)
Supplement: Supplementary file 1 [file tropicalmed-11-00193-s001.zip › tropicalmed-4356664-supplementary.pdf]

Article

Tuberculosis-Related Hospitalization According to Comorbidity Burden: A Retrospective Single-Center Cohort Study

Supplementary Table S1. Distribution of individual Charlson Comorbidity Index comorbidities among patients with tuberculosis

Comorbidity	Number (%)
Myocardial infarction	3 (3.70)
Congestive heart failure	6 (7.41)
Peripheral vascular disease	3 (3.70)
CVA or TIA	13 (16.05)
Dementia	3 (3.70)
Chronic pulmonary disease	6 (7.41)
Connective tissue disease	5 (6.17)
Peptic ulcer disease	0
Liver disease (mild)	4 (4.94)
Liver disease (moderate to severe)	3 (3.70)
Diabetes mellitus (uncomplicated)	23 (28.40)
Diabetes mellitus (end-organ damage)	4 (4.94)
Hemiplegia	2 (2.47)
Moderate to severe CKD	8 (9.88)
Solid tumor (localized)	6 (7.41)
Solid tumor (Metastatic)	5 (6.17)
Leukemia	1 (1.23)
Lymphoma	0
AIDS	0

CVA, cerebrovascular accident; TIA, transient ischemic attack; CKD, chronic kidney disease; AIDS, acquired immunodeficiency syndrome

Supplementary Table S2. Methods of tuberculosis diagnosis

	Number (%)
Microbiological confirmation	61 (75.31)
Histopathological confirmation	6 (7.41)
Clinical and radiological confirmation	14 (17.28)
